# Additive Manufacturing of β-NiAl by Means of Laser Metal Deposition of Pre-Alloyed and Elemental Powders

**DOI:** 10.3390/ma14092246

**Published:** 2021-04-27

**Authors:** Michael Müller, Bastian Heinen, Mirko Riede, Elena López, Frank Brückner, Christoph Leyens

**Affiliations:** 1 Department of Additive Manufacturing and Printing, Fraunhofer Institute for Material and Beam Technology, Winterbergstraβe 28, 01277 Dresden, Germany; bastian.heinen@iws.fraunhofer.de (B.H.); mirko.riede@iws.fraunhofer.de (M.R.); elena.lopez@iws.fraunhofer.de (E.L.); frank.brueckner@iws.fraunhofer.de (F.B.); christoph.leyens@iws.fraunhofer.de (C.L.); 2 Faculty of Mechanical Science and Engineering, Institute of Materials Science, Dresden University of Technology, Helmholtzstr. 7, 01069 Dresden, Germany; 3 Department of Engineering Sciences and Mathematics, Luleå University of Technology, 971 87 Luleå, Sweden

**Keywords:** additive manufacturing, laser metal deposition, nickel aluminide, intermetallics, advanced materials

## Abstract

The additive manufacturing (AM) technique, laser metal deposition (LMD), combines the advantages of near net shape manufacturing, tailored thermal process conditions and in situ alloy modification. This makes LMD a promising approach for the processing of advanced materials, such as intermetallics. Additionally, LMD allows the composition of a powder blend to be modified in situ. Hence, alloying and material build-up can be achieved simultaneously. Within this contribution, AM processing of the promising high-temperature material β-NiAl, by means of LMD, with elemental powder blends, as well as with pre-alloyed powders, was presented. The investigations showed that by applying a preheating temperature of 1100 °C, β-NiAl could be processed without cracking. Additionally, by using pre-alloyed, as well as elemental powders, a single phase β-NiAl microstructure can be achieved in multi-layer build-ups. Major differences between the approaches were found within substrate near regions. For in situ alloying of Ni and Al, these regions are characterized by an inhomogeneous elemental distribution in a layerwise manner. However, due to the remelting of preceding layers during deposition, a homogenization can be observed, leading to a single-phase structure. This shows the potential of high temperature preheating and in situ alloying to push the development of new high temperature materials for AM.

## 1. Introduction

### 1.1. NiAl-Based Alloys

Within the last few decades, aero engines have been steadily developed in order to increase efficiency and fulfil required maintenance intervals. In the course of rising inlet temperature, state-of-the-art nickel-based superalloys, such as Inconel 718 or Mar-M 247, have reached their limits. Hence, new high-temperature materials are essential to meet rising material requirements [1,2].

The intermetallic β-NiAl phase exhibits a low density of only two thirds of comparable Ni-based superalloys. Moreover, the high melting temperature of 1638 °C, superior thermal conductivity and an outstanding oxidation resistance at elevated temperatures make β-NiAl-based alloys promising materials for high-temperature applications in future turbine engines, which has been known since the 1960s (Table 1) [2,3,4,5,6,7]. However, the unsolved issues of limited ductility and fracture toughness at room temperature, as well as a poor strength at high temperatures, have prevented its transfer to industry so far [2,3]. Especially, comparison of polycrystalline β-NiAl to the Ni-based superalloy René 80 underlines the drawbacks regarding the yield strength of β-NiAl alloys at the current state of development [8].

The limited ductility and poor fracture toughness of β-NiAl are caused by its cubic B2 structure, exhibiting only three independent slip systems at room temperature and therefore not fulfilling von Mises criterion [8]. Overcoming these challenges using manufacturing and alloying technology could yield a tremendous mass reduction of turbine rotors of up to 40% [8].

### 1.2. Laser Metal Deposition

**Figure 1:**
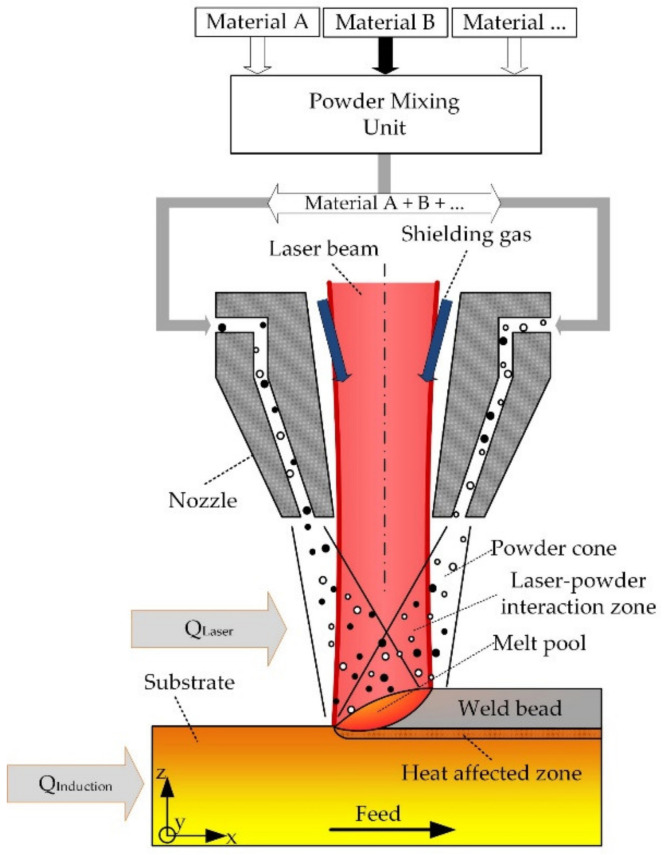
Illustration of the multi-material LMD process with additional inductive substrate heating, reproduced and adapted with permission from [10].

Laser metal deposition (LMD, also referred to as direct energy deposition) is a well-known process for the coating and refurbishment of parts, and has recently gained a great deal of attention regarding its application as an additive manufacturing (AM) technology [9,10,11,12]. The basic principle of LMD is depicted in Figure 1.

Powdery base material is delivered into the process by a carrier gas. In the process zone, the powder is subsequently pre-heated by a laser and finally absorbed by the laser-induced melt pool. By the relative movement between the substrate and the deposition head, three-dimensional structures can be manufactured [13]. Furthermore, the blown powder approach enables the simultaneous feeding of different powders into the process zone. Hence, an in situ alloying and an in situ adaption of alloy compositions can be achieved [14]. Additionally, the LMD process can be combined with auxiliary energy sources (e.g., inductive heating) for tailoring thermal boundary conditions, such as cooling rates and the overall process temperature [13].

Thus, LMD combines the advantages of near net shape manufacturing, a high freedom regarding the tailoring of thermal process conditions and the possibility of in situ alloying. Therefore, the processing of advanced materials, which often require elevated process temperatures or adapted cooling rates is very promising and was already investigated by various researches [13,15,16,17,18].

### 1.3. Laser Metal Deposition of Ni-Al-Alloys

Due to the advantages of LMD, various researchers have investigated the capabilities of this technology for processing Ni-Al alloys with different chemical compositions [19,20,21,22,23,24,25,26,27]. Kotoban et al. [19,20,21] investigated the processing of pre-alloyed Ni85Al15 powder material by LMD for single- as well as multi-layer build-ups. For the investigations, a 2 kW Yb:YAG disk laser was used and a spot diameter of 2 mm, a laser power of 400 W and feeding speeds between 0.83 and 10 mm/s were applied. The investigations showed the formation of multiple phases in the deposited material despite the stoichiometric composition of the powder material. Moreover, a defect free processing of multi-layer build-ups could not be achieved.

Abboud et al. [26] as well as Liu et al. [22] applied an in situ mixing of elemental powders to manufacture Ni-Al alloys with different compositions. By mixing elemental Al and Ni single phase β-NiAl alloys, as well as multi-phase alloys consisting of β-NiAl, γ-Ni, γ’-Ni_3_Al and Ni_2_Al_3_, were manufactured. By adjusting the powder feeding rates of Ni and Al, both groups of researchers were capable of controlling the emerging metallurgical phases. At a substrate heating temperature of 500 °C, a reduction of cracking susceptibility was observed enabling the crack free deposition of an eight-layer functionally graded cuboid specimen. However, within these studies, neither the reproducibility of the in situ alloying process nor the fabrication of single-phase cuboid specimens was investigated.

Liu et al. [24] investigated the in situ formation of a Ni-Al intermetallic compound with added phosphorous by laser melting of blended powder placed on a Ni-alloy substrate. XRD analysis of single weld beads showed the presence of the β-NiAl, γ-Ni, and γ’-Ni_3_Al phases. By adding phosphorus, a refinement of the microstructure could be achieved. In addition, results from Kramer et al. [27] showed the feasibility of in situ alloying of Ni-Al-alloys by LMD. Within their work, the manufacturing of a γ’-Ni_3_Al cuboid specimen was achieved.

The reviewed literature shows the great potential of LMD for processing Ni-Al-alloys. However, there is still a gap regarding in-depth work on the defect free manufacturing of β-NiAl-based-alloys. Within this contribution, a first step towards closing this gap shall be taken by investigating the processing of pre-alloyed powder, as well as elemental powder blends for manufacturing single phase β-NiAl build-ups. This work shall serve as a baseline investigation for further development of NiAl-based alloys that can be beneficially processed by LMD.

## 2. Materials and Methods

Within the presented work pre-alloyed Ni50Al50 powder, as well as elemental Al (purity >99.9%) and Ni (purity >99.9%) (Nanoval GmbH & Co. KG, Berlin, Germany), were processed for manufacturing β-NiAl specimens. For process development and sample manufacturing the following steps were conducted:

I. Characterization of powder and substrate material,
II. Deposition of single-tracks,
III. Deposition of multi-track build-ups,
IV. Deposition of single-layers,
V. Deposition of multi-layer build-ups.

Deposition was conducted with a COAX14 coaxial powder nozzle from Fraunhofer IWS (Fraunhofer IWS, Dresden, Germany) and a LDF 1500–400 diode laser from Laserline GmbH (Laserline GmbH, Mülheim-Kärlich, Germany) with a maximum power of 1.5 kW and a spot diameter of 1.6 mm. For all trials, Ar was used as the carrier gas and the shielding gas. To assure reproducible process conditions, the nozzle measurement system LIsec (Fraunhofer IWS, Dresden, Germany) developed by Fraunhofer IWS was used for powder cone characterization.

**Figure 2:**
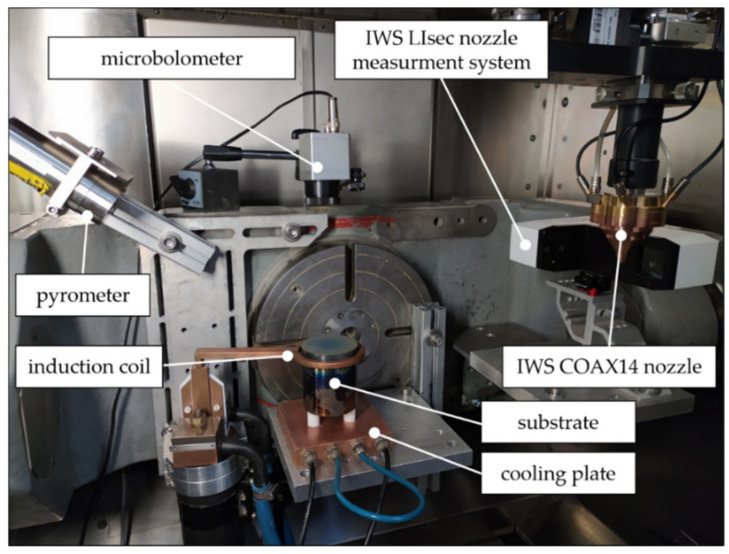
Test set up for the conducted LMD trials.

According to investigations from Zeumer [28], β-NiAl exhibits a brittle-to-ductile-transitions-temperature (BDTT) between 600 and 900 °C, depending on the strain rate. Therefore, inductive heating enabling substrate temperatures of over 1100 °C was applied. Substrate temperature measurement and control could be achieved using a laterally installed pyrometer and an infrared camera. The complete process set up is shown in Figure 2. The main experimental equipment is listed in Table 2. In the following, the process development is described in detail.

### 2.1. Powder and Substrate Material

The pre-alloyed Ni50Al50 powder, as well as the elemental Ni and Al powders, were produced by inert gas atomization. All powders exhibited a nominal Gaussian particle size distribution between 50 and 150 µm. The powders were characterized by light optical microscopy (LOM) (Olympus K. K., Tokio, Japan), scanning electron microscopy (SEM) (Jeol, Tokio, Japan) and atomic emission spectroscopy (ICP-OES) (Bruker, Billerica, MA, USA) with respect to morphology, particle size, porosity and chemical composition. The substrate material was Ni-alloy 201 with a nominal purity of 99.6%. Additionally, the substrate material was analyzed by ICP-OES. The ICP-OES analysis was conducted for the base materials Ni and Al and the most common technically relevant contaminations.

**Figure 3:**
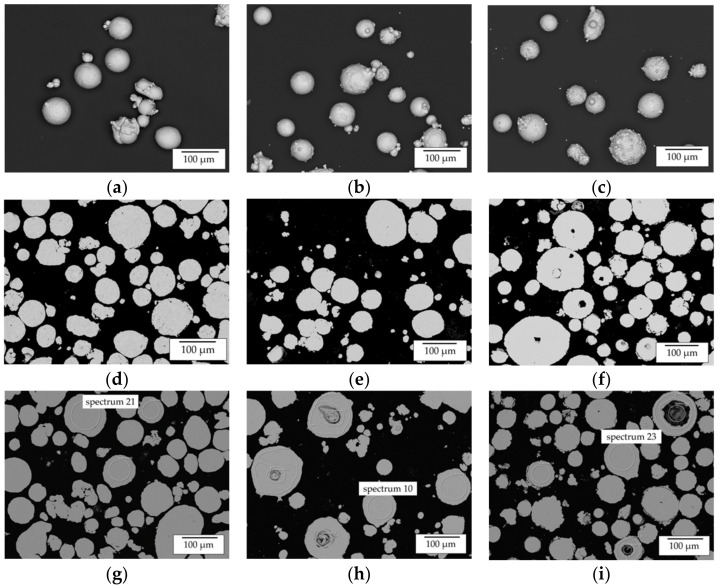
Overview of SEM images of the used powder material—(**a**–**c**) SEM of loose powder particles (**a**—Al, **b**—Ni; **c**—NiAl); (**d**–**f**) SEM of powder particle cross sections (**d**—Al, **e**—Ni; **f**—NiAl); (**g**–**i**) SEM of powder particle cross sections with marked regions, which were analyzed by EDX (**g**—Al, **h**—Ni; **i**—NiAl).

The results of the chemical analyses of the used powder material are listed in Table 3. An overview of SEM images showing lose powder and particle cross sections of the processed Al, Ni and Ni50Al50 is given in Figure 3. Based on these images, EDX measurements and geometrical investigations of the powder particles were carried out. In order to assure a stable in situ alloying process similar geometrical characteristics of the processed powder are expected to be beneficial.

### 2.2. Single-Track Investigations

For the pre-alloyed and elemental powders, single-tracks were manufactured and characterized with respect to the formation of pores and cracks, the degree of dilution and the track shape.

Single-tracks with a length of 20 mm were fabricated using the sets of process parameters listed in Table 4 (Set 1–5). All tracks were welded with a constant powder mass flow of 2 g/min and constant carrier and shielding gas flow.

Based on the parameters developed for processing pre-alloyed Ni50Al50 powder material single-tracks were manufactured by blending elemental Al and Ni. Due to the flammability of pure Al, the welding trials were only conducted without pre-heating. The fabrication was conducted using the parameters presented in Table 4 (Set 6). The elemental powders were fed with a total powder mass flow of 2 g/min at an atomic ratio of 1:1.

### 2.3. Multi-Track Investigations

Parameter Set 5 (see Table 4) was selected for multi-track manufacturing. By applying three consecutive single-tracks with alternating welding directions wall structures could be fabricated.

According to multi-track manufacturing with pre-alloyed powder three consecutive single tracks with alternating welding directions were deposited using the parameter shown in Table 4 by blending elemental Ni and Al. As for the single-track trials, in situ synthesis was only applied without substrate heating.

### 2.4. Single- and Multi-Layer Investigations

**Figure 4:**
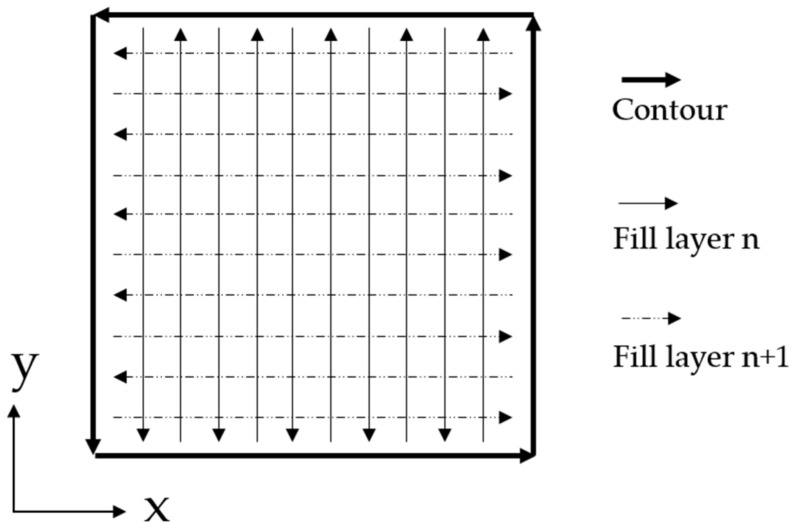
Weld path pattern for single-layers and multi-layer build-ups.

In accordance with the single-track trials, single-layers and multi-layer build-ups made of 5 to 20 layers were fabricated in order to investigate the influence of accumulating stress on the formation of defects. Hence, the sets of parameters 1 to 6 shown in Table 4 were used. The welding strategy depicted in Figure 4 (Set 6) was applied. The weld tracks were divided into fill and contour tracks. Between every adjacent track as well as between contour and fill, an overlap of 50% of the track width was applied. After every layer, the weld path pattern was rotated by 90° to increase the samples isotropy.

For in situ syntheses by mixing elemental powders, only multi-layer build-ups were investigated in detail. The welding strategy was identical to processing of the pre-alloyed powder and the set of parameters shown in Table 4 (Set 6) was applied.

## 3. Results

In the following, the results of the powder characterization as well of the manufacturing trials are presented. Sample characterization and analysis were conducted by LOM, SEM, EDX and XRD analysis. The results of the material characterization are discussed in detail.

### 3.1. Powder Analysis

**Figure 5:**
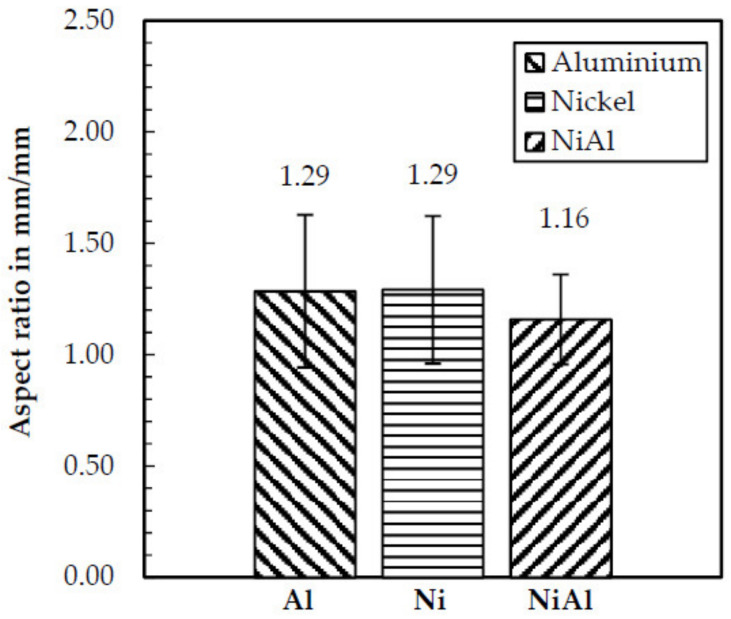
Aspect ratio of the used powders determined by image analysis.

SEM analysis of all three powders revealed a high degree of satellites and irregular particle shapes. Using image analysis, the median aspect ratio was calculated. The results in Figure 5 show a lack of sphericity of all powders, indicated by an aspect ratio between 1.16 and 1.29; however, this was not expected to influence the welding process negatively, since a stable powder feeding could be achieved during powder mass flow tests and powder cone measurements (carried out using a LIsec measurement system).

**Figure 6:**
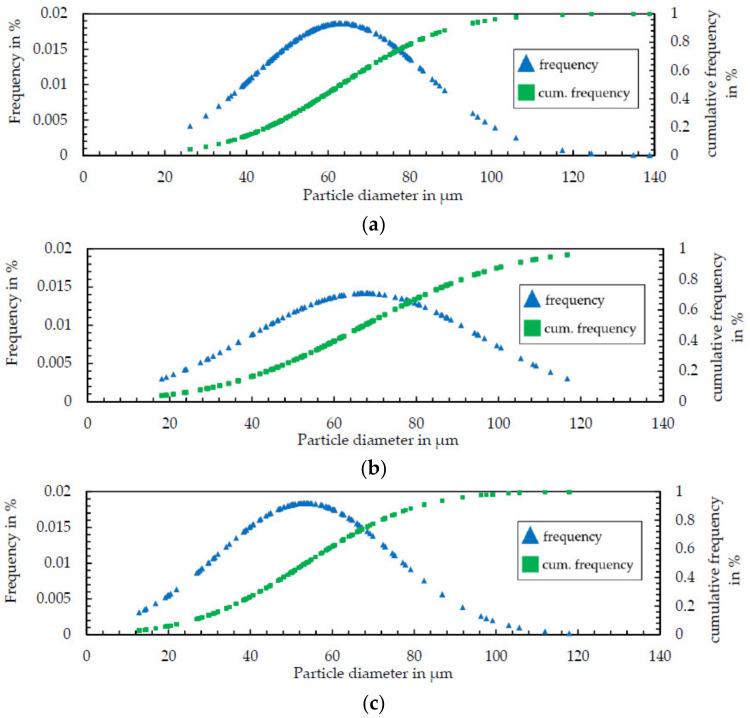
Particle size distribution of the used Ni50Al50 powder (**a**), Al powder (**b**) and Ni-alloy 201 powder (**c**) determined by image analysis.

Additionally, the accordance of aspect ratios of the elemental powders might positively influence the in situ alloying process. Furthermore, also based on image analysis, the particle size distribution was calculated. The results for all powders are shown Figure 6 (a—Ni50Al50, b—Al, c—Ni). All powders exhibited a median particle diameter (d_50_) between 52 and 67 µm with a standard deviation between 21 and 28 µm. This similarity of particle shapes for all powders is considered sufficient to assure comparability between the processing of pre-alloyed and elemental powder blends and is expected to ensure stable powder flow during in situ synthesis.

**Figure 7:**
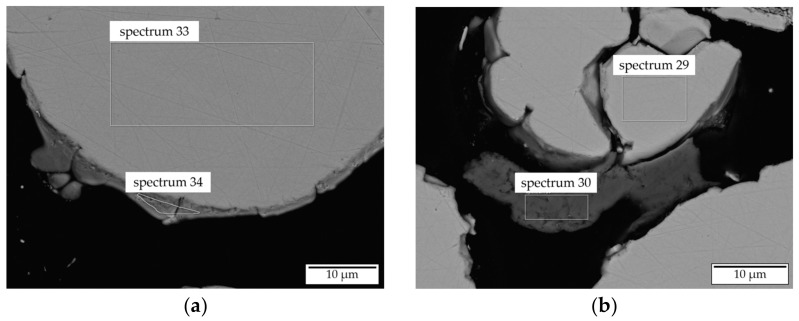
SEM images of Ni50Al50 powder cross sections with the marked regions investigated by EDX ((**a**)—region 33 and 34; (**b**)—region 29 and 30).

In addition to morphology analysis, the chemical composition of all powders was investigated by ICP-OES and EDX. The results are listed in Table 3 and Table 5. The corresponding regions of interest investigated by EDX are marked in the images shown in Figure 3g–i and Figure 7. Based on these images, EDX measurements and geometrical investigations of the powder particles were carried out. In order to assure a stable in situ alloying process, similar geometrical characteristics of the processed powder are expected to be beneficial. The EDX analysis of the elemental powders corresponds well to the nominal composition and the ICP-OES analysis as no contamination could be detected.

EDX analysis of the Ni50Al50 powder material revealed the presence of three different regions within the powder particles. Region 1 (spectra 23, 29 and 33), the bulk of the powder particles, exhibited the expected nominal chemical composition with a maximum deviation of 1.6 at.%.

According to the Ni-Al-phase diagram, these deviations in chemical composition are not critical to the formation of a single-phase β-NiAl structure. Region 2 (spectrum 30) and region 3 (spectrum 34) show the formation of layers consisting of elemental Al/Ni on the surface of the Ni50Al50 powder particles. The formation of these layers is expected to be caused by evaporation phenomena during the atomization process. However, due to the overall composition determined by ICP-OES, the presence of the elemental layers is also not considered critical for the manufacturing of single-phase β-NiAl structures.

### 3.2. Single-Tracks

**Figure 8:**
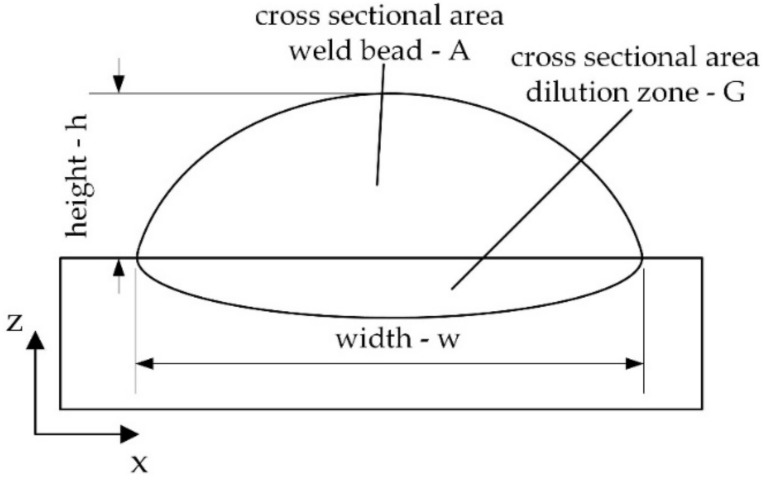
Geometrical features of single LMD weld tracks.

In order to evaluate the used parameters with respect to applicability for multi-layer build-ups the LOM images of the cross sections were analyzed with respect to shape (Figure 8), defects, as well as the microstructure within the track–substrate interface.

(1) $$a=\frac{h}{w}$$

(2) $$d=\frac{G}{A+G}$$

where *a*—aspect ratio; *h*—height, *w*—width; *d* = dilution; *G* = cross sectional area dilution zone; and *A*—cross sectional area dilution zone.

#### Geometrical Analysis

**Figure 9:**
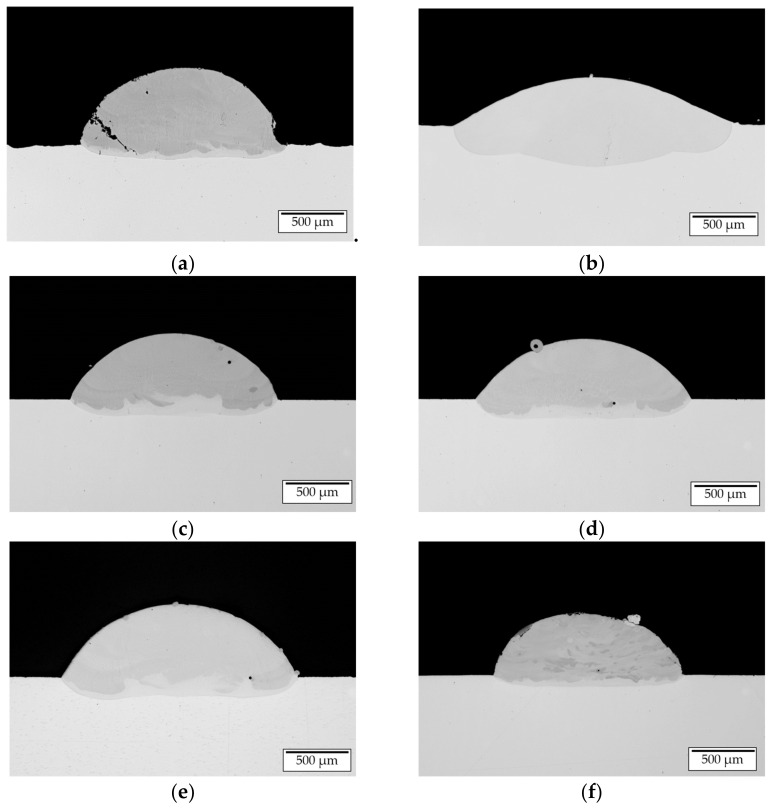
LOM images of cross sections of manufactured single tracks (**a**)—set 1, (**b**)—set 2, (**c**)—set 3, (**d**)—set 4, (**e**)—set 5, (**f**)—set 6.

To evaluate the used parameters with respect to applicability for multi-layer build-ups the LOM images of the cross sections (Figure 9) were analyzed by image analysis using the freely accessible software ImageJ (Version 1.52, Rasband; Bethesda, MD, USA).

The investigated geometrical features height, width, weld bead cross section and dilution zone cross section are illustrated in Figure 8. Based on these geometrical features the characteristics aspect ratio *a* and the degree of dilution *d* were determined (see formula 1 and 2). The results are presented in Table 6. According to Riede et al. [29], an aspect ratio between 0.3 and 0.5 is recommended to ensure accuracy and high build-up rates during multi-layer manufacturing. For pre-alloyed powder processing and in situ synthesis parameters for achieving an aspect ratio of approx. 0.3 and a degree of dilution as low as 0.2 at room temperature could be developed (Sets 1 and 6). Increasing the substrate temperature at constant process parameters yields a tremendous flattening of the weld bead due to the additional energy input by the induction system. This causes a drop of the aspect ratio to 0.17 and an increase of the dilution zone cross section leading to a degree of dilution of 0.53. Moreover, an increase of the melt pool temperature is expected. Hence, a compensation of increased substrate and melt pool temperature needs to be taken into consideration. By decreasing the laser power by 47% at all preheating temperatures (700, 800 and 900 °C) tracks with sufficient aspect ratios of approx. 0.3 were manufactured. With rising pre-heating temperature, an increase in degree of dilution was observed.

#### Defect Analysis

**Figure 10:**
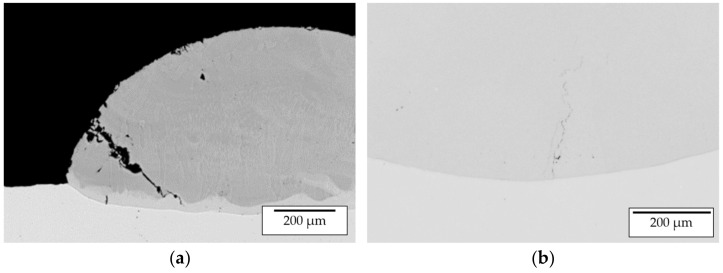
(**a**) Cold cracking due to processing without substrate pre-heating; (**b**) hot crack formation within single track.

The analysis of occurring defects within the single tracks showed the formation of two types of cracking. Single-tracks manufactured without pre-heating exhibit severe cold cracking (Figure 10a) due to manufacturing below the BDTT, which is expected to be approx. 500–900 °C for β-NiAl depending on the strain rate [28].

Single tracks manufactured at pre-heating temperature above 700 °C do not show any cold cracking phenomena. However, within the track-substrate interface very fine cracking can be observed (Figure 10b). These cracks are considered to be hot cracks caused by supercooled regions during solidification. Since this type of cracking was only observed for one set of parameters with an excessive energy input and this type of cracking is not considered as a major challenge in processing single phase β-NiAl no further analysis of this hot cracking was conducted.

#### Microstructural Analysis

**Figure 11:**
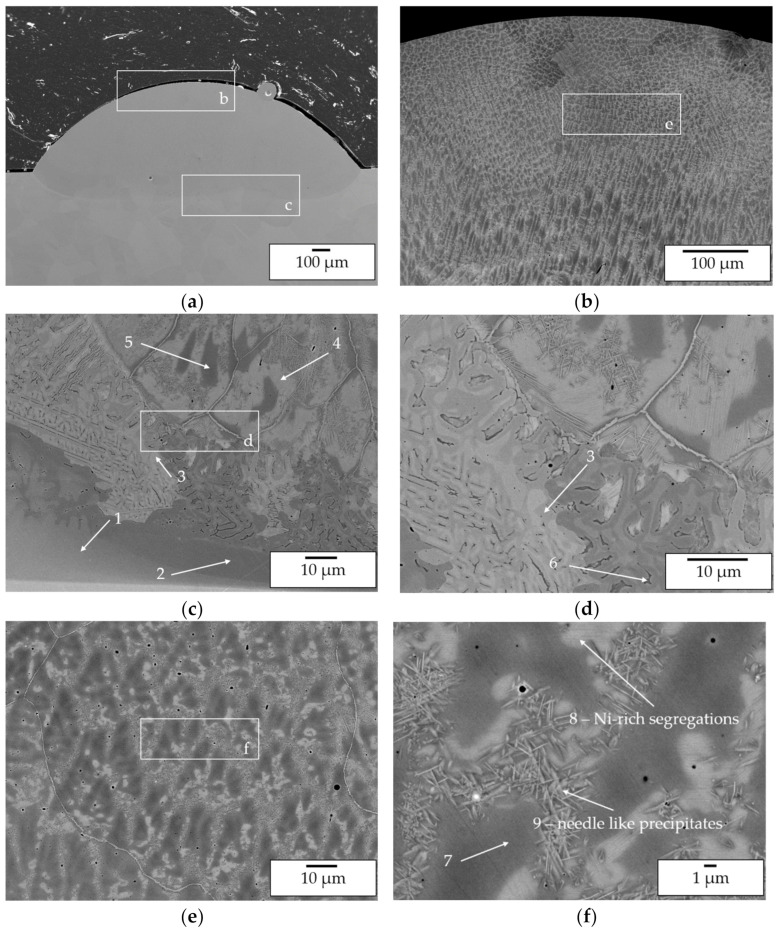
SEM images of a weld track deposited with parameter set 4; (**a**) overview of the weld track; (**b**) top region of weld track at 200× magnification; (**c**) transition region from substrate to weld track at 100× magnification with marked regions 1–5 investigated by EDX; (**d**) transition zone from substrate to weld track at 2000× magnification with marked region 3 and 6 investigated by EDX; (**e**) top region of weld track at 1000× magnification; (**f**) top region of weld track at 5000× magnification with marked regions 7–9 investigated by EDX.

For microstructure formation during single-track manufacturing at elevated temperatures, parameter Set 4 (900 °C pre-heating temperature) was analyzed by SEM (Figure 11). For phase evaluation, selected areas were analyzed by EDX (Table 7). The selected regions are marked in Figure 11c,d,f. These analyzed regions reveal the formation of several phases differing from the expected β-NiAl. This is expected to be caused by a large difference in density and melting temperatures of the processed materials, leading to a high degree of intermixing of the Ni substrate and the processed powder.

During solidification, the cooling rate G decreases with increasing distance from the substrate. Thus, the solidification morphology varies over the weld bead cross section. This phenomenon can also be observed within the analyzed weld bead. A transition from columnar to equiaxed grain structure can be seen in Figure 11b. Within the transition zone, (Figure 11c,d) different phases with varying Al content ranging from 17.1 up to 36.6 at.% were identified. Hence, the formation of the phases γ-Ni, γ’-Ni_3_Al, β-NiAl as well as Ni_5_Al_3_ is expected.

Within the top zone (Figure 11f) two phases (regions 7/8 and 9) could be recognized. The matrix consists of 37.5 at.% Al and is expected to be β-NiAl with a reduced Al content caused by the rapid solidification. Within this matrix nickel-rich segregation with 35.9 at.% Al can be observed forming a cellular structure. Along the cellular nickel-rich segregations, a nickel-rich phase with needle-like morphology was found. According to results from Cui et al. [30], gathered within reaction synthesis investigations with elemental Ni and Al, the needle-like structure could be identified as γ’-Ni_3_Al.

**Figure 12:**
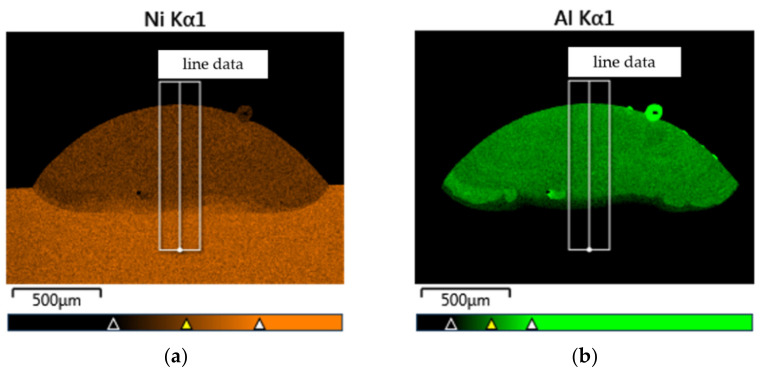
EDX mapping of the Ni (**a**) and as well as Al (**b**) content within of a weld track deposited at 900 °C pre-heating temperature, 800 W laser power and 400 mm/min feeding speed.

**Figure 13:**
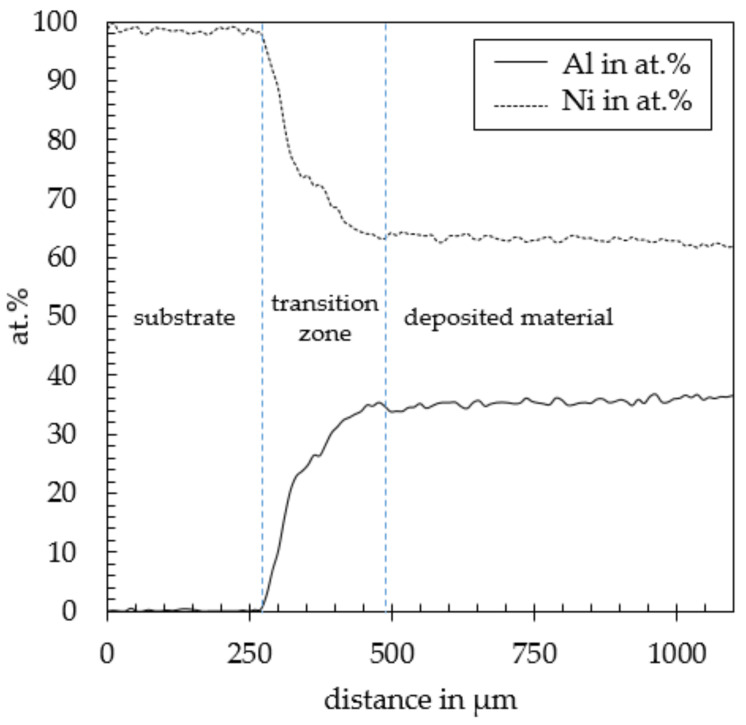
EDX data along the weld track showing the high degree of dilution in weld track deposited at 900 °C pre-heating temperature, 800 W laser power and 400 mm/min feeding speed.

By EDX the overall composition of the weld track along the center line (Figure 12) was investigated. The evaluation of this data in Figure 13 reveals a large gap between the chemical composition of the processed powder and the deposited track.

The variation of chemical composition can be divided into three regions. Region 1 exhibits a nickel content of above 99.9 at.% and represents the uninfluenced substrate. Following region 2, the dilution zone, with a depth of approximately 250 µm and a varying Ni content from 99.9 at.% down to approx. 64 at.%. At 64 at.% Ni region 3 with a stable chemical composition begins. In comparison to the chemical composition of the processed powder material a drop of the aluminum content by approx. 11 at.% can be observed yielding the described shift of phases. On the one hand, this shift in chemical composition might be caused by the high degree of dilution. On the other hand, an aluminum loss due to evaporation of low melting phases can be considered another reason.

**Figure 14:**
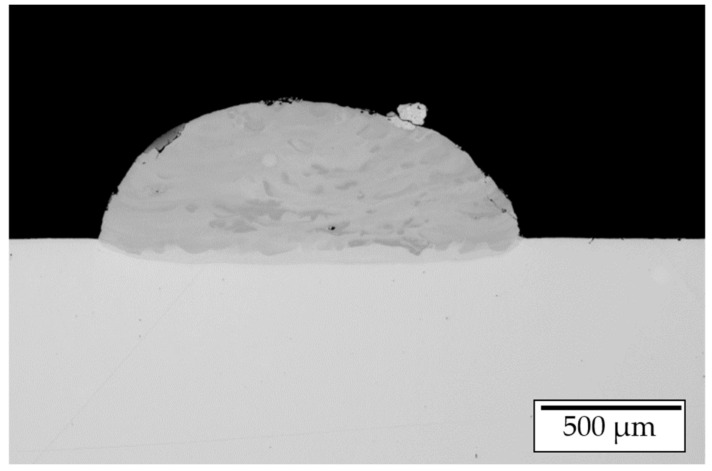
LOM image of a single track manufactured by in-situ alloying of elemental Ni and Al.

Single tracks were manufactured by simultaneous feeding of elemental Ni and Al (elemental ratio n(Ni)/n(Al) = 1). For manufacturing, a laser power of 1250 W and a feeding speed of 400 m/min were applied. A light optical microscopy image of a single-track cross section is shown in Figure 14. The image shows an inhomogeneous elemental distribution in a layer wise manner over the cross section.

Additionally, a slight asymmetric shape of the weld track can be detected. The lack of mixing is expected to be caused by the varying melting temperatures, the severely different densities and the melt pool convection in combination with a varying viscosity of the melt pool. Similar observations were made by Gasper et al. [31] within studies on in-situ alloying titanium aluminides.

In order to evaluate elemental mixing and phase formation during in-situ alloying microstructure investigation was conducted on a multi-track wall specimen (see Section 3.3). This enables the investigation of the elemental mixing with and without the influence of the substrate material.

### 3.3. Multi-Track Build-Ups

**Figure 15:**
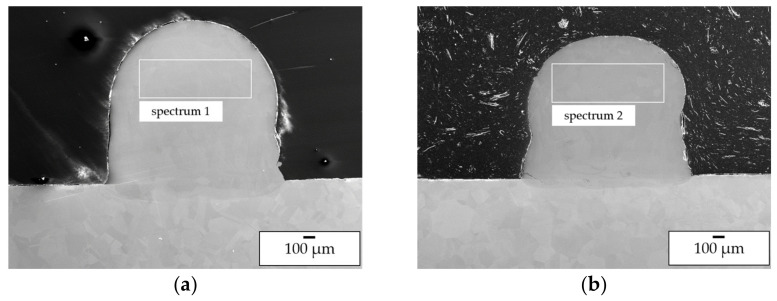
SEM BSE images of wall specimens consisting of three tracks manufactured using pre-alloyed powder (**a**) and in situ alloying (**b**).

In order to investigate the elemental mixing in the transition zone from substrate to deposited material as well as in the deposited material without substrate dilution three-layer wall specimens were manufactured and investigated by SEM and EDX. The images are shown in Figure 15. Processing of the pre-alloyed powder was conducted using a pre-heating temperature of 1100 °C (parameter set 5). In-situ alloying was conducted at room temperature (parameter set 6).

#### Microstructure Analysis

Firstly, the investigation of the elemental composition in the top layers by EDX (spectra 1 and 2 in Figure 15) showed that for both processing approaches a suitable Ni/Al ratio for the formation of β-NiAl could be achieved (pre-alloyed powder 52.8 5 at.% Ni/47.2 at.% Al; in-situ alloying: 53.7 at.% Ni/46.3 at.% Al). A comparison of the elemental composition of the pre-alloyed feedstock and the deposited material shows that no Al loss has occurred. Hence, the reduced Al content observed within the single-track investigation is associated with the intermixing of the layer with the Ni substrate material.

**Figure 16:**
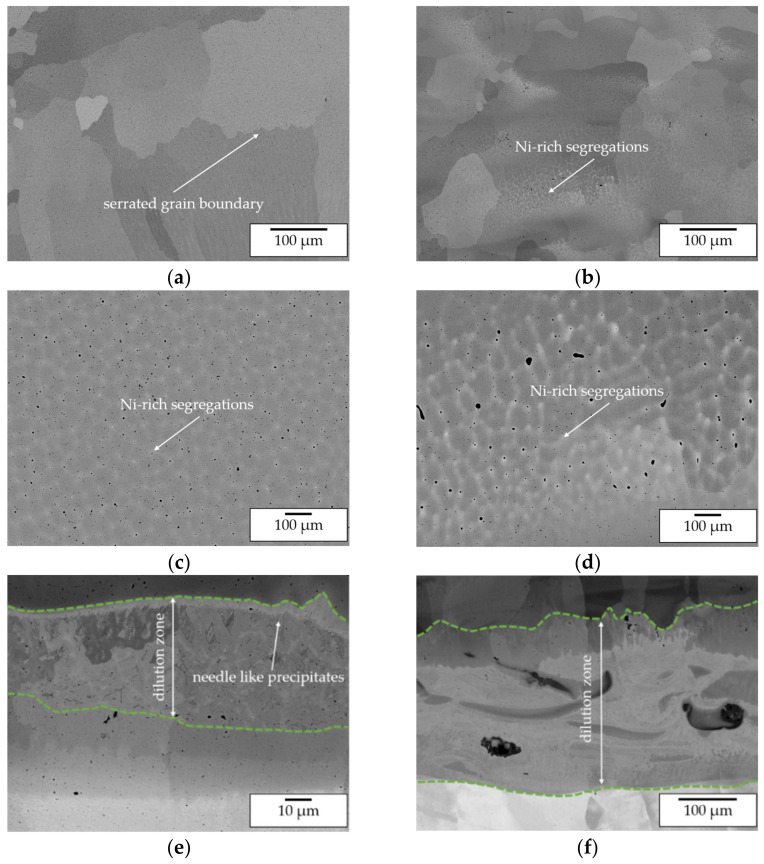
SEM images of manufactured wall specimens—(**a**) top layer, pre-alloyed powder processing; (**b**) top layer, in situ alloying; (**c**) Ni-rich segregations, pre-alloyed powder processing; (**d**) Ni-rich segregations, in situ alloying; (**e**) transition zone, pre-alloyed powder processing; (**f**) transition zone, in situ alloying.

Secondly, SEM images of the top layers (Figure 16) reveal that for both approaches a single phase β-NiAl structure was formed. Differences can be observed with respect to the formation of cellular Ni-rich segregations within the β-NiAl grains. For pre-alloyed powder processing at elevated temperatures uniformly distributed segregations were found. For in situ alloying, the segregations are distributed inhomogeneously so that even grains without segregations are being formed.

**Figure 17:**
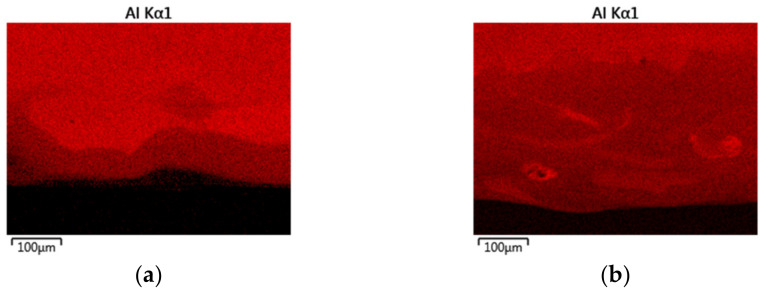
EDX mapping of the Al distribution within the transition zone showing the differences in intermixing between the processing of pre-alloyed (**a**) and elemental (**b**) powders.

This indicates a superior homogenization of the microstructure due to the high process temperatures as well as the processing of pre-alloyed powder. Furthermore, major difference between the processing approaches were found regarding the substrate near dilution. For pre-alloyed powder processing a transition zone width of approx. 50 µm was determined. Within this zone Ni grains with needle-like precipitates are formed. As already stated for the single-track analysis these precipitates are expected to be Ni_3_Al. For in-situ alloying a larger transition zone width of approx. 300 µm was measured. Within this zone a severe lack of intermixing and the formation of a layerwise structure with varying Ni/Al content can be observed. This corresponds to lack of intermixing that was already found within the in situ alloying of single-tracks and is caused by the high gap in density and melting temperature of Ni and Al. The EDX mapping of Al in Figure 17 underlines the described differences. In conclusion, it can be stated that, without taking the dilution zone into consideration, the processing of elemental powders is suitable for in situ alloying of β-NiAl and delivers a similar microstructure compared with the conventional processing of pre-alloyed powder.

### 3.4. Single Layers and Multi-Layer Build-Ups

**Figure 18:**
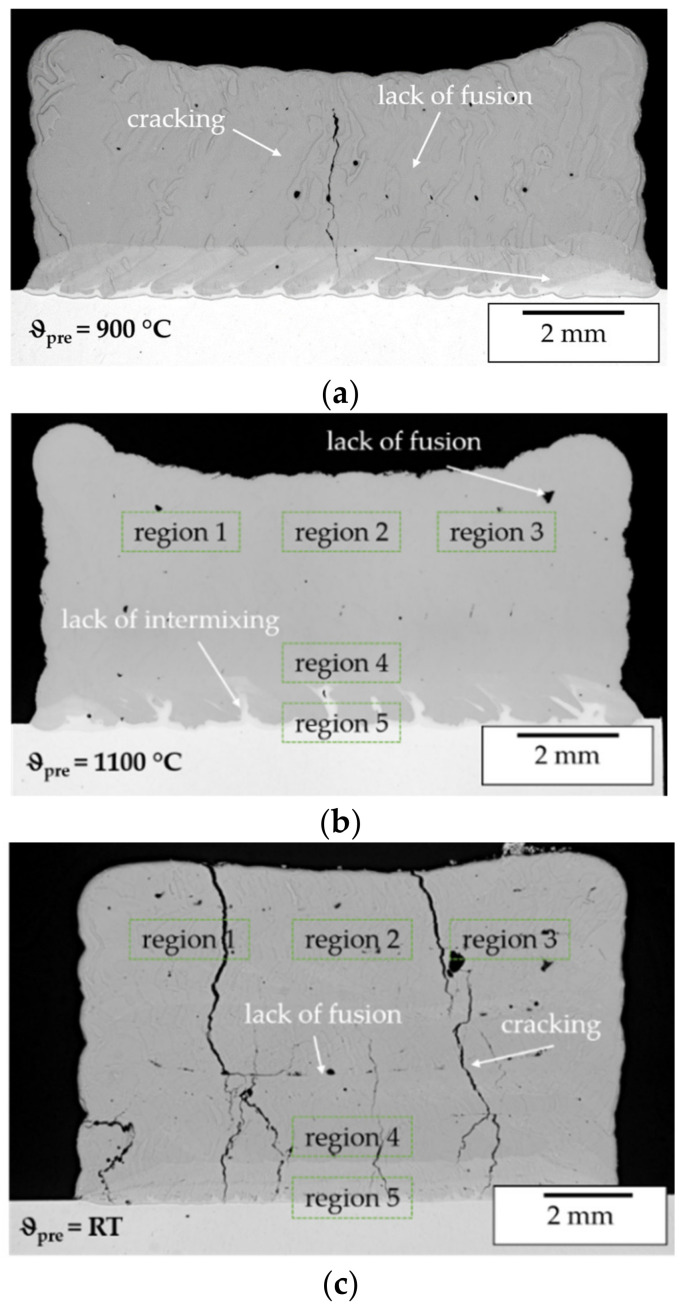
LOM images of cuboid specimens manufactured using pre-alloyed powder ((**a**) preheating temperature 900 °C; (**b**) preheating temperature 1100 °C) and in situ alloying ((**c**) no preheating), marked regions indicate areas analyzed by XRD.

Based on the parameters listed in Table 4 and the bidirectional welding strategy single layers and cuboid specimens (Figure 18) were manufactured using pre-alloyed powder and in situ alloying.

For pre-alloyed powder, specimens were manufactured at varying pre-heating temperatures ranging from RT to 1100 °C. For in situ alloying, only RT manufacturing was investigated. For sample characterization LOM, SEM, EDX and XRD, as well as computed tomography (CT), were applied.

#### Microstructure and Defect Analysis

LOM images of cross section reveal crack formation up to a pre-heating temperature of 900 °C (see Figure 18a) for specimens manufactured using pre-alloyed powder and by in situ alloying.

**Figure 19:**
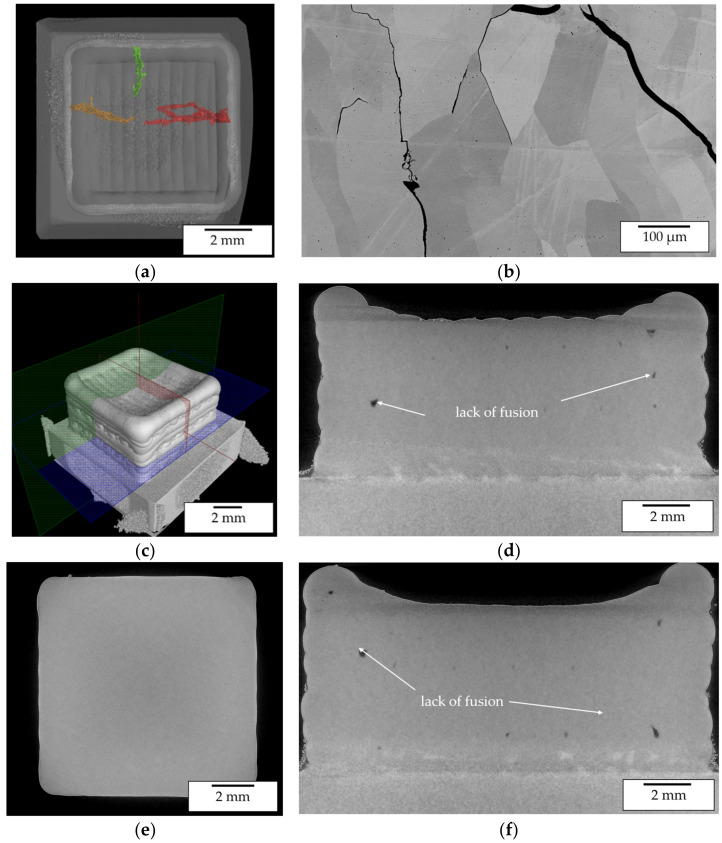
(**a**) CT image exhibiting crack formation perpendicular to the alternating welding direction in a cuboid specimen manufactured at 900 °C preheating temperature using pre-alloyed powder; (**b**) SEM image of crack propagation within cuboid specimen; (**c**) CT iso-view image of cuboid specimen; (**d**) CT image of cross section in zx-plane; (**e**) CT image of cross section in xy-plane; (**f**) CT image of cross section in zy-plan.

The cracks form vertically and perpendicularly to the welding direction and propagate through several layers as was observed by LOM and CT imaging as is shown in Figure 19a. Furthermore, SEM imaging shows that the cracking mainly occurs intergranularly as can be seen in Figure 19b. This indicates that the cracking was induced by excessive strain during welding while not exceeding the BDTT. Hence, due to the high strain rates during solidification the BDTT is not exceeded at a temperature of 900 °C.

The analyses of a cuboid specimen processed at a preheating temperature of 1100 °C showed that at this pre-heating temperature cracking can be eliminated. Neither in LOM images of cross sections (Figure 18b) nor in CT images (Figure 19c–f) cracking could be observed. Hence, at 1100 °C the BDTT is exceeded and a crack free processing of the pre-alloyed β-NiAl powder material can be achieved. For all samples intertrack and interlayer defects could be observed.

**Figure 20:**
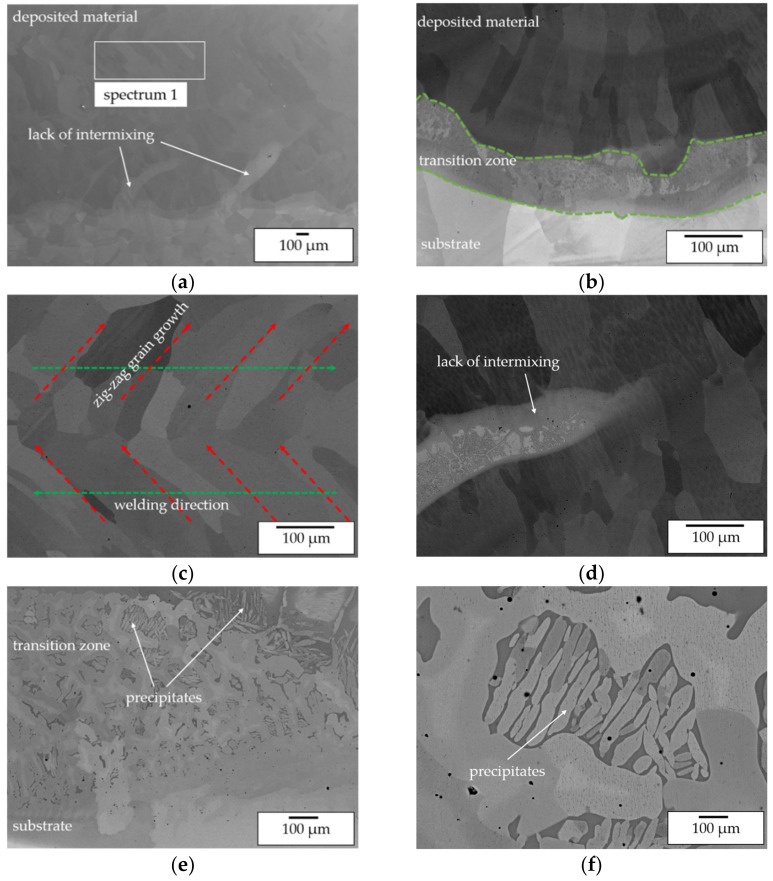
SEM BEC images of a cuboid specimen manufactured with pre-alloyed powder at 1100 °C pre-heating temperature—(**a**) overview showing transition from Ni substrate to the deposited single phase β-NiAl material; (**b**) Ni-NiAl transition zone at 200× magnification; (**c**) zig-zag grain structure of the deposited single phase β-NiAl material; (**d**) lack of intermixing causing multi-phase Ni-rich regions; (**e**) transition zone at 1000× magnification; (**f**) transition zone at 500× magnification showing the formation of precipitates.

**Figure 21:**
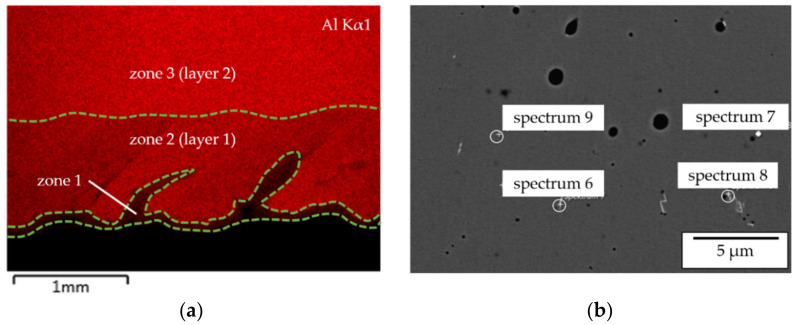
(**a**) EDX mapping of the Al distribution within the transition zone; (**b**) EDX analysis of Zr-rich precipitates within the deposited material.

**Figure 22:**
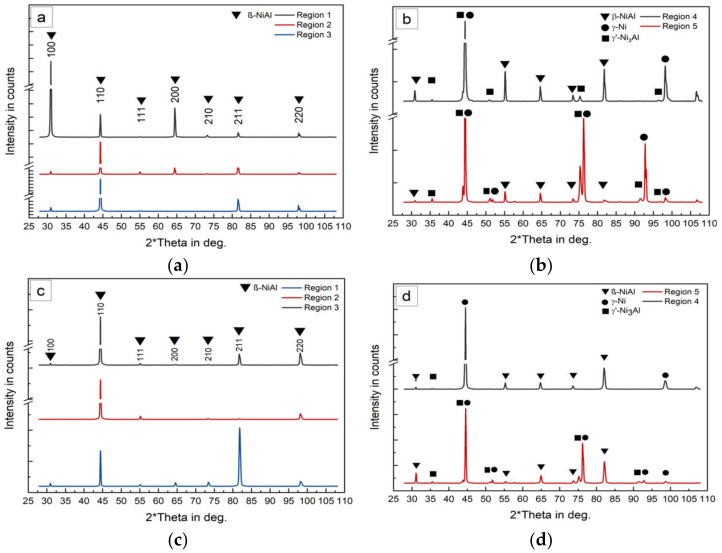
XRD patterns in different regions of a cuboid specimen manufactured using pre-alloyed powder (**a**,**b**) and in situ alloying (**c**,**d**), reference in Figure 14.

These defects are mostly caused by a lack of fusion. During the processing of β-NiAl the high thermal conductivity and the high melting temperature required a high energy input to achieve sufficient melting of powder and substrate. Hence, defect elimination could probably be achieved by an increase in laser power. This will be investigated in further trials. The crack free specimen manufactured at 1100 °C was further investigated by SEM, EDX (Figure 20 and Figure 21) and XRD (Figure 22). The results confirm the findings from the previous multi-track specimen analysis.

Within regions free from substrate dilution, a single phase β-NiAl structure formed as can be seen in Figure 20a–c. The presence of only β-NiAl was confirmed by XRD investigations. The results are presented in Figure 22 and the investigated regions are marked in Figure 18b. In all three regions (1–3), only peaks caused by the β-NiAl phase could be identified.

The β-NiAl grain structure and orientation was influenced by the welding strategy leading to a zig-zag grain structure (Figure 20c), as the grain growth follows the thermal gradient during solidification. Moreover, within the β-NiAl grains Zr-rich precipitates in nm scale were identified by EDX (Figure 21b, Table 8). Due to the distance to the substrate of the investigated region the Zr is expected to be a minor contamination of the powder feedstock. The influence of this Zr-rich precipitates on the mechanical properties should be analyzed in further investigations.

The transition zone from substrate to the deposited material is characterized by two phenomena. On the one hand, the overall transition zone from Ni to β-NiAl exhibits a small width of only 100 µm (Figure 20b and Figure 21a, zone 1). A local lack of intermixing can be observed, which leads to small regions with a locally increased Ni content (and Figure 21a, zone 1).

On the other hand, the EDX mapping in Figure 21a shows that within the first layer of deposition (zone 2) a reduced overall Al content occurs, which is likely to be also caused by dilution. Beginning with the second layer (zone 3) the feedstock composition as measured in spectrum 1 in Figure 20a is reached.

In order to analyze the phase formation during in situ alloying of multi-layer build-ups the specimen depicted in Figure 18c was analyzed in the marked regions by XRD. The results are presented in Figure 22c,d and match the results for pre-alloyed powder processing.

As described for the multi-track trials, this lack of intermixing is likely to be caused by the large difference in density on elemental Ni, Al and NiAl, as well as the gaps in melting temperature. Within both kinds of regions, a multi-phase structure was observed. XRD analysis (Figure 18b, regions 4 and 5) of the transition zone suggests that the precipitates found by SEM imaging (Figure 20) represent the Ni_3_Al phase.

For the transition zone from substrate to deposited material (Figure 18b,c, regions 4 and 5) similar XRD patterns were obtained for pre-alloyed powder processing and in situ alloying. In both cases the peaks indicate the presence of β-NiAl, γ’-Ni_3_Al as well as γ-Ni. Within regions (Figure 18b,c, regions 1–3) that are not influenced by the dilution of the substrate, only β-NiAl peaks were detected indicating that a single phase β-NiAl was synthesized successfully by mixing the elemental Ni and Al powders. The cracking during in situ alloying caused by processing temperatures below the BDTT will be overcome in the future by combining inductive heating and LMD in inert gas atmosphere. This enables the preheating of reactive materials without an increased risk of inflammation.

## 4. Summary and Outlook

Due to the low density, a high melting temperature and superior thermal conductivity β-NiAl based alloys are potential materials to replace current state-of-the-art nickel-based superalloys. However, low ductility and fracture toughness at room temperature as well as limited high temperature strength in a binary state have prevented its transfer into industrial applications so far. Within this contribution, first steps towards near net shape additive manufacturing of β-NiAl based alloys, as well as in situ alloy modification, were successfully taken. Within the presented work, pre-alloyed Ni50Al50 as well as elemental Ni and Al powders were processed by LMD in order to additively manufacture single phase β-NiAl structures. For overcoming manufacturing challenges, such as crack formation, the innovative approach of energy source coupling (laser and inductive heating) was applied. The investigations delivered the following major results:

1. By energy source coupling (laser and inductive heating) single-tracks, layers as well as cuboid specimens (10 × 10 × 5 mm^3^) could be successfully manufactured without cracking by processing pre-alloyed powder. For crack free fabrication of cuboid specimens, a pre-heating temperature of 1100 °C had to be applied. Defect analysis was conducted by means of LOM and CT. Microstructure analysis by SEM and XRD revealed the formation of β-NiAl, γ-Ni, as well as γ’-Ni_3_Al within single tracks and in the dilution zone of manufactured multi-track and cuboid specimens due to a high degree of dilution. Starting at the second layer the samples exhibit a single phase β-NiAl microstructure.
2. Single tracks and layers as well as cuboid specimens were successfully manufactured by in-situ mixing elemental Ni and Al powders. Since no pre-heating was applied due to flammability of elemental Al, no crack free specimens could be manufactured by in-situ alloying. However, XRD and SEM analysis of wall and cuboid specimens showed that by in situ mixing a single phase β-NiAl microstructure could be achieved. By the simultaneous use of separate powder feeders, the Ni/Al ratio could be tailored to form the desired phase. Within the first layer a severe lack of intermixing could be observed leading to high elemental gradients and inhomogeneous phase formation within the dilution zone. This inhomogeneous transition zone is bigger compared to the pre-alloyed powder-based process. Due to a remelting of previous tracks and layers a homogenization takes place during deposition, which diminishes the lack of intermixing and supports the homogenous formation of the desired phase.
3. The feasibility study showed that by energy source coupling single phase β-NiAl can be processed by LMD without cracking. Furthermore, the in situ alloying investigations showed that this technique can be successfully applied to in situ form single phase β-NiAl by mixing elemental Ni and Al. Inhomogeneous phase formation and lack of intermixing can only be observed within the substrate-layer transition zone.

Based on the very promising results gathered, further steps towards the processing of more complex β-NiAl based alloys can be taken. For this, the approach of energy coupling as well as in situ alloying for rapid alloy development can be applied to tailor the alloy according to application and also process-related requirements.

## Figures and Tables

**Table 1:** Comparison of state-of-the art nickel-based superalloy IN 718 and β-NiAl [3,4].

Properties	β-NiAl	IN 718
density (g·cm^−3^)	5.9	8.2
therm. conductivity (W·m^−1^·K^−1^)	76.0	11.4
melting range (°C)	1638	1260–1336
ϑ_max_ creep resistance (°C)	1100	700
ϑ_max_ oxidation resistance (°C)	1400	1000

**Table 2:** Equipment used for the presented experimental investigations.

Equipment	Type
Laser source	Laserline LDF 1500–400, Germany
Deposition system	Fraunhofer IWS COAX 14, Germany
Powder feeder	GTV PF 2/2, Luckenbach, Germany
Induction system	EMAG eldec MICO 50/80, Dornstetten, Germany
IR-Camera	Optris PI 640, Berlin, Germany
Pyrometer	Advanced Energy Impac IGA 6/23, Frankfurt, Germany
Light Optical Microscope (LOM)	Olympus GX51, Japan
Scanning Electron Microscopy (SEM)	Jeol JSM 6610, Japan
Energy-dispersive X-ray spectroscopy (EDX)	Oxford instruments X-MAX, Abingdon, UK

**Table 3:** Chemical composition of the powder and substrate material.

Alloy	Ni (at.%)	Al (at.%)	Fe (at.%)	Si (at.%)	Mn (at.%)
Ni	99.92	0.00	0.042	0.032	0.003
Al	0.00	99.9	0.019	0.015	0.001
Ni50Al50	52.86	47.0	0.031	0.024	0.002
Alloy 201	99.82	0.00	0.042	0.032	0.099

**Table 4:** Overview of the process parameters and pre-heating temperature for the analyzed single tracks made of pre-alloyed powder. (RT—room temperature).

Set	Laser Power (W)	Feeding Speed (mm/min)	Temperature (°C)
1	1500	400	RT
2	1500	400	700
3	800	400	700
4	800	400	900
5	800	400	1100
6	1250	400	RT

**Table 5:** Elemental composition of selected powder particles.

Spectrum	Ni (at.%)	Al (at.%)	Si (at.%)
10	98.3	-	-
21	-	98.5	-
23	50.8	49.2	-
29	51.6	48.4	-
30	1.0	94.31	-
33	51	49	-
34	94.6	1.4	0.3

**Table 6:** Geometrical features of the deposited tracks for the selected sets of parameters.

Set	w (mm)	h (mm)	A	G	a	d
1	1.74	0.58	0.73	0.16	0.33	0.18
2	2.34	0.39	0.57	0.63	0.17	0.53
3	1.62	0.52	0.63	0.17	0.32	0.21
4	1.67	0.56	0.69	0.19	0.34	0.22
5	1.8	0.52	0.65	0.27	0.29	0.29
6	1.66	0.52	0.67	0.16	0.31	0.19

**Table 7:** Regions analyzed by EDX within a single track manufactured at 900 °C pre-heating temperature, 800 W laser power and 400 mm/min feeding speed, reference Figure 11.

Region	Spectrum	Ni (at.%)	Al (at.%)
Transition zone	1	82.9	17.1
	2	75.8	24.2
	3	78.4	21.6
	4	64.3	35.7
	5	63.4	36.6
	6	64.0	36.0
Top zone	7	62.5	37.5
	8	64.1	35.9
	9	65.7	34.3

**Table 8:** Chemical composition of the analyzed region marked in Figure 21.

Spectrum	Ni (at.%)	Al (at.%)	Zr (at.%)
6	49.9	32.9	17.2
7	50.1	36.4	13.4
8	48.9	43.4	16.7

## Data Availability

The data presented in this study are available on request from the corresponding author.
